# Short term memory for serial order: unraveling individual differences in the use of processes and changes across tasks

**DOI:** 10.3389/fpsyg.2013.00589

**Published:** 2013-09-13

**Authors:** Gabriela V. Koppenol-Gonzalez, Samantha Bouwmeester, Jeroen K. Vermunt

**Affiliations:** ^1^Department of Methodology and Statistics, Tilburg UniversityTilburg, Netherlands; ^2^Department of Psychology, Erasmus University RotterdamRotterdam, Netherlands

**Keywords:** short term memory, memory for serial order, verbal and visual processes, similarity effect, individual differences, latent class analysis

## Abstract

In this study we investigated whether we could distinguish the use of specific verbal and visual short term memory (STM) processes in children, or whether the differences in memory performance could be interpreted only in terms of quantitative differences. First, the number of processes involved in the responses on six STM tasks (serial order reconstruction) of 210 primary school children aged 5–12 years was examined by means of latent states. The number of items to reconstruct was manipulated to unravel quantitative differences in responses (high or low performance), and the similarity of the items was manipulated to distinguish qualitative differences in responses (verbal or visual processing). Furthermore, we examined how children changed from one type of process to another on tasks with list lengths of 3, 5, and 7 items by means of the dynamics between the latent states using a latent Markov model. The results showed that two latent states representing the use of specific verbal and visual STM processes could be distinguished on all the tasks. Moreover, two latent states showing merely differences in performance were also found. These findings underline the value of latent variable models to unravel differences between as well as within individuals in the use of cognitive processes.

## Introduction

Short term memory (STM) processes have been the focus of cognitive studies for a long time and many researchers have found considerable differences in performance on verbal and visuospatial STM tasks in adults (Baddeley, 1986, 2000; Logie et al., 2000; Miyake et al., 2001) as well as in children (Hitch et al., 1989; Gathercole et al., 2004; Kane et al., 2004). It has long been assumed, therefore, that STM consists of qualitatively distinct verbal and visuospatial processes. However, this assumption has been questioned by researchers arguing that the differences in STM performance actually reflect differences in task demands and procedures between verbal and visuospatial tasks, rather than differences in memory processes (Avons, 1998; Avons and Mason, 1999; Ward et al., 2005). Yet others have stated that there is no distinction between verbal and visuospatial STM processes, but that memory should be considered as a modality-independent unitary system (Jones et al., 1995; Chuah and Maybery, 1999).

In the current study, we unravel the types of memory processes that children show on STM tasks for serial order, taking task conditions and individual differences into consideration. These are issues that are often not taken into account when investigating STM in children, but they may influence the responses of the children considerably. Therefore, we want to account for individual differences in the use of STM between children as well the influence of task demands on STM performance of children. Specifically, we examine whether the differences in memory processes between children can be interpreted in terms of qualitatively distinct verbal and visual processes, and we explore the changes in the use of memory processes within children as the task demands change. Although adults have also been found to show variation in their responses due to individual differences in the use of STM processes (e.g., Logie, 1995), we focus on memory performance of children. Due to developmental differences, children show larger variation in their responses than adults, allowing us to test specific hypotheses about the meaning of such variation. For instance, we can test whether there will be an increase in performance as well as changes in the use of verbal and visual STM as children get older (Gathercole et al., 1994; Gathercole and Pickering, 2000; Alloway et al., 2006) or whether there will only be better overall performance, due to the development of one common underlying system, as children get older (Chuah and Maybery, 1999). In either case, it is important to take the task demands of verbal and visual STM tasks and their influence on performance into account (Avons, 1998; Avons and Mason, 1999; Ward et al., 2005).

The theory most often referred to by modality-specific models is the model of working memory of (Baddeley and Hitch, 1974; Baddeley, 1986). In this model, the central executive is a modality-independent, attention-based system with limited capacity, which controls two modality-specific slave systems, referred to as the phonological loop and the visuospatial sketchpad. These systems represent STM, which serves to store information. The phonological loop is used for storage and processing of verbal information, whereas the visuospatial sketchpad is used for storage and processing of visual and spatial information (Baddeley, 2000). Empirical support for the two distinct slave systems for verbal and visuospatial information in the Baddeley and Hitch model of WM is provided especially by studies using a latent variable approach (Miyake et al., 2001; Kane et al., 2004; Alloway et al., 2006). In these studies participants are typically presented with different verbal and visuospatial WM tasks and with different verbal and visuospatial STM tasks. The results of confirmatory factor analyses typically show that the best fitting models represent a modality-independent WM system and two modality-specific STM systems.

According to other researchers, however, the differences between verbal and visuospatial STM tasks do not allow a direct comparison between verbal and visuospatial STM processes. Avons (1998) noted that the tasks used in most studies on verbal STM tap memory for serial order and the stimuli most often used are familiar verbal material (e.g., words). The tasks used in most studies on visuospatial STM, however, tap item recognition of novel visual material (e.g., patterns, matrices). As a consequence, when the scores on each serial position are plotted in a graph, the serial position curves of verbal STM tasks differ from those of visuospatial STM tasks. In a series of experiments, Avons (1998) showed that the results typically found with familiar verbal material, tapping memory for serial order, can also be found with familiar visual material when implemented into a task that also taps memory for serial order. In another study, Ward et al. (2005) demonstrated that the results typically found with novel visual material, tapping item recognition, can also be found with novel verbal material when the task used also taps item recognition. These researchers suggest that the verbal/visual distinction in STM processes has been overvalued and that the real distinction is that of memory for item vs. order information (however, see Purser and Jarrold, 2005).

There have been adult studies on memory for serial order measuring verbal and visuospatial STM with equivalent tasks. The conclusions about underlying verbal/visual processes were therefore based on the same task conditions across modalities and can be attributed with more certainty to the actual memory processes used. Saito et al. (2008) investigated order recall and modality-specific interference on performance. Their results showed that visual processing was used in conditions impairing verbal processing, as expected, but also that visual processing was still present in the different conditions allowing verbal processing. The overall conclusion was that visual STM processes were used to support order recall of verbal information, and therefore that verbal and visual processes can be considered as distinct systems, even under the same task conditions (Saito et al., 2008).

Guérard and Tremblay (2008), also keeping task conditions equal, examined serial position curves of verbal and spatial STM tasks by conducting an in-depth analysis of errors on different serial positions, assuming that specific types of errors (i.e., omissions, transpositions, and intrusions) and serial position curves are related to specific STM processes (see Henson, 1998). The first experiment of Guérard and Tremblay showed that the distribution of overall errors was similar for the verbal and spatial tasks and that this was also true for almost all of the specific types of errors that were analyzed. In their second experiment, the authors additionally examined the influence of verbal and spatial interference by administering a secondary suppression task during the verbal and spatial STM tasks and found that the effect of this interference on performance was modality-specific.

Unfortunately, the results between the two studies mentioned above are not quite comparable, because the tasks used to measure memory for serial order differ between the two studies. Guérard and Tremblay (2008) used serial order reconstruction tasks, whereas Saito et al. (2008) used serial order recall tasks. Furthermore, the type of manipulations to investigate the effect of modality-specific interference also differs. Guérard and Tremblay (2008) used verbal and spatial suppression, whereas Saito et al. (2008) used phonological and visual similarity of their stimuli. These subtle differences in task demands (similarity or suppression and order recall or reconstruction) may have had differential influences on responses of different individuals in the studies of Guérard and Tremblay and Saito and colleagues. We learned from these studies in the first place that it is rather crucial for unraveling and examining individual differences in the use of STM processes to use a task design in which performance can fairly be compared in terms of visual and verbal processing. Below we explain how we dealt with these design issues.

It has been shown that similarity of the items to be recalled is a useful manipulation of stimuli to distinguish the use of verbal and visual STM processes (Conrad, 1971; Hitch et al., 1989; Logie, 1995; Logie et al., 2000; Baddeley, 2003; Poirier et al., 2007). When items that are visually similar are recalled worse than items that are visually dissimilar this indicates the use of visual processes, that is, when items are memorized based on their visual features and those visual features are similar, the visual similarity causes confusion in visual STM when the items have to be immediately recalled in their correct serial order. In comparison, when the visually similar items are memorized based on their verbal labels, the visual similarity does not cause confusion in verbal STM because the verbal labels do not sound similar. Conversely, when items that are phonologically similar are recalled worse than items that are phonologically dissimilar this indicates the use of verbal processes according to the same principle: the phonological similarity causes confusion when the items are memorized based on their verbal labels. If phonologically similar items are memorized visually, the phonological similarity does not cause confusion in visual STM.

The effect of similarity on performance is not only dependent on the type of materials used, but on individual differences in the use of STM processes as well. For instance, verbal material such as words has been shown to be susceptible to visual similarity effects, because words can also be processed visually (Logie et al., 2000; Saito et al., 2008) and this additional visual processing of verbal material has been shown to be adopted by some individuals but not by others (Logie et al., 1996).

These individual differences in processing can be inferred from the specific similarity effects on performance. Typically, the effects of similarity are compared to a control condition, in which the stimuli are phonologically and visually dissimilar. Verbal STM processes are reflected by significantly lower performance on phonologically similar stimuli than on control stimuli (i.e., phonologically dissimilar). Visual STM processes are reflected by significantly lower performance on visually similar stimuli than on control stimuli (i.e., visually dissimilar). The use of both verbal and visual STM processes is characterized by significantly lower performance on both the phonologically and the visually similar stimuli than on control stimuli, and the use of neither verbal nor visual STM processes is characterized by no differences between the phonologically and visually similar, and the control stimuli.

When a suppression task is used as interference, individual differences in processing are harder to investigate. All the participants are then presented with a secondary task during the memory task to prevent the use of verbal or visual processing and therefore, all the participants are forced to use the same type of processing. In comparison, manipulating the similarity of stimuli allows the participants to use either verbal or visual processing, but then the similarity of the items will affect their performance in a specific way, which in turn allows us to infer the type of processing used by each individual. Therefore, we used similarity of the stimuli as interference instead of modality-specific suppression.

The effect of similarity is detrimental on performance only when memory for serial order is required, as opposed to memory for item information which leads to better performance when the items are similar (Tehan et al., 2001; Baddeley, 2003; Fallon et al., 2005; Chasse and Belleville, 2009). Therefore, it is important to use a memory task that specifically measures memory for serial order. A serial order recall task is suitable for measuring the effects of similarity only when combined with an item recall task. Otherwise, the effect of similarity on order recall is confounded by the effect on item recall (Nairne and Kelley, 2004). After all, in serial order recall tasks participants are asked to recall items in their correct position. Therefore, a serial order reconstruction task which requires a participant to reconstruct the serial order of items that are given is more appropriate to investigate the effects of similarity on memory for serial order of certain stimuli than a serial order recall task.

Another reason to use serial order reconstruction tasks instead of serial order recall tasks is that the responses on the latter tasks are verbal that is, either written or spoken. This is problematic, because if we want to unravel verbal and visual STM processes, the participant should be able to use either verbal or visual processing. Using a task that requires a verbal response makes it impossible for participants to use solely visual processing, because they are forced by the task demands to process verbally at output. In a serial order reconstruction task, the participant is required to reconstruct the serial order that was presented without having to give a verbal response. An additional advantage is that when no written response is required, these tasks can easily be administered to young children (see e.g., Majerus et al., 2006). This way, children of a wide age range can be presented with the same tasks and procedures, keeping the task conditions equal across children.

In a previous study, we (Koppenol-Gonzalez et al., 2012) manipulated the inter-item similarity of STM tasks that could be processed verbally as well as visually (see Hitch et al., 1989 and Poirier et al., 2007 for a similar approach). Accounting for the differential effects of similarity on order recall tasks, the children were presented with order recall and item recall tasks. Also, the tasks consisted of three and five items to recall. On the easier task with three items, half of the children showed visual processing and on the harder task with five items, most of the children showed verbal processing. Task difficulty seemed to affect the children's responses more than child characteristics such as age. In the current study we administered serial order reconstruction tasks instead of item and order recall, so that the task conditions of the phonologically and visually similar tasks would be more equivalent, tapping memory for serial order information and not requiring a verbal response. This way, we could verify whether we would still find a distinction in responses between children in terms of verbal and visual processes. This verification was tested against the hypothesis that there is no such distinction when the task conditions are kept constant. Furthermore, we added a difficulty level to the tasks in order to investigate how individual children change their use of STM processes as the list length of the tasks increases.

To address our aim of unraveling the types of memory processes that children show on STM tasks for serial order, we first defined the expected effects of similarity within individuals on their performance and what it means in terms of STM processes. To examine whether there was a distinction in the use of visual and verbal STM processes, the scores on a task with visually similar items and the scores on a task with phonologically similar items were compared to each other. Because we were mainly interested in those individuals who used *only* visual or *only* verbal STM processes, we compared the scores of the tasks with a certain list length within individuals. The use of verbal processes was expected to be reflected by significantly lower scores on the phonologically similar task compared to the visually similar (i.e., phonologically dissimilar) task, indicating susceptibility only to phonological similarity and not to visual similarity. The use of visual processes was expected to be reflected by significantly lower scores on the visually similar task compared to the phonologically similar (i.e., visually dissimilar) task, indicating susceptibility only to visual similarity and not phonological similarity. In comparison, if the scores on the phonologically and visually similar task would be equal, we would not be able to draw conclusions about verbal or visual processing. There are several reasons why performance would be affected in the same way by visual and phonological similarity, such as a combination of verbal and visual processes being used, or the existence of one general memory system in which no specific verbal or visual processes are used. Therefore, we only drew conclusions about the use of specific verbal or visual STM processes when a clear difference between phonological and visual similarity was found.

To address our aim of examining whether the differences in memory processes between children can be interpreted in terms of qualitatively distinct verbal and visual processes, we investigated whether there were subgroups of children in our sample showing different effects of similarity. If all the children would use the same type of processing, the effects of similarity would be the same for all children and there would be no subgroups, but just the sample. This would support the view of one general STM system (Jones et al., 1995). If there are two or more subgroups in our sample, the interpretation of the types of processes used in each subgroup depends on the effects of similarity. If the effect of similarity is the same in all the subgroups and the distinction between the subgroups is only quantitative (only differences in overall performance), this would also support the view of one modality-general STM system (Chuah and Maybery, 1999). If the effect of similarity in one subgroup is opposite to the effect of similarity in the other subgroup, this would mean that some children are susceptible only to phonological similarity while others are susceptible only to visual similarity. This would lead us to interpret the first group as children who used verbal processing and the second group as children who used visual processing during the memory tasks.

To address our aim of exploring the changes in the use of memory processes within children as the task demands change, we used tasks with list lengths of 3, 5, and 7 items. We suggest that if a child shows for instance, verbal processing on one task that child would not necessarily show verbal processing on subsequent tasks with increasing difficulty as well. Therefore, we also examined whether the specific STM processes a child shows initially would change as the list length of the subsequent tasks increases. If children use the same processes irrespective of the number of items they have to recall, the use of STM processes can be considered to be a stable child characteristic, which may reflect for instance a certain developmental stage (Gathercole et al., 2004). However, adult studies show differences in type of processing across task conditions (Logie et al., 2000). Therefore, in the current study, we focused not only on the distinction between verbal and visual processes, but on the dynamics between STM processes as list length increased from 3 to 5 up to 7 items as well.

## Methods

### Participants

The participants were 210 children aged 4;11 (4 years, 11 months) to 12;9 years (*M* = 8;8 years, *SD* = 2;2 years, 110 girls). The children were selected from kindergarten through the sixth grade of two different primary schools in an urban area of the Netherlands. From each grade, 30 children were selected. The schools where the children were selected from have an agreement with Erasmus University Rotterdam for research purposes; the children from these schools already had permission to participate in studies conducted by researchers from Erasmus University. The agreement is approved by the Ethics Committee of Psychology (ECP) at the Erasmus University Rotterdam, the Netherlands. The ECP concluded that a formal approval of the committee was not required for each individual study, because the participants were given full-disclosure of the procedure (i.e., there was no deceit), the experimental procedure was non-invasive and the results were analyzed anonymously. Furthermore, the participants were citizens from the Netherlands who were recruited via their schools and participated voluntarily. Before starting the data collection, all the parents were informed about this particular study by writing. From the group of children with parental permission, 210 children were randomly selected. After participation, all the children received a small reward.

### Materials

The sequences that had to be reconstructed in their correct serial order consisted of pictures of simple nameable line drawings. Therefore, the children could process the pictures visually and/or they could process the names of the pictures verbally. Half of the pictures were visually similar and represented words that were phonologically dissimilar (see also Hitch et al., 1989 and Poirier et al., 2007) and half of the pictures were visually dissimilar and represented words that were phonologically similar. Whether children actually used the labels we intended for each picture, was tested in a pilot study (*N* = 14). None of these children were included in the current sample. The pictures that were labeled with other (phonologically dissimilar or long) names during the pilot were removed or replaced for the experimental tasks. We chose to pilot the labels that children would use for the pictures instead of practicing the labels prior to the experiment, because we did not want to impose verbal processing.

Visual similarity was reflected by pictures with similar outlines and angles of orientation. For instance, a visually similar sequence contained pictures that were all triangles, but some details made the first triangle represent a tent, while the second triangle represented a sailing boat, the third a mountaintop, the fourth a roof top, and the fifth a pointed cap. Phonological similarity was reflected by pictures which' names were end-rhyme words, such as *rat-cat-mat-hat-bat* (Conrad, 1971). The words used in this study were actually Dutch rhyme words.

Besides the similarity of the (names of the) pictures, the task conditions were held as constant as possible. For instance, most pictures represented one-syllable words, but in some conditions it was not possible for all the sequences to consist of only one-syllable words. In those cases, we matched the phonologically similar sequence with a visually similar sequence that consisted of words with the same amount of syllables. This way, we kept difficulty in terms of word-length constant over the visually and phonologically similar tasks with the same list lengths.

Finally, the different tasks contained sequences of 3, 5, or 7 pictures, which were either visually or phonologically similar. Specifically, the children were presented with six tasks and a total of 30 pictures; a sequence of 3 phonologically similar pictures, a sequence of 3 visually similar pictures, a sequence of 5 phonologically similar pictures, a sequence of 5 visually similar pictures, a sequence of 7 phonologically similar pictures, and a sequence of 7 visually similar pictures. All the items, pictures and names with translations, are given in Appendix A.

### Procedure

The computerized tasks were administered to the children individually on a 15.4-inch laptop in a quiet room in school. Before each task was administered, all the children received a practice trial of the same list length as the experimental trial. The stimuli of the practice trials were visually dissimilar pictures with phonologically dissimilar names. The children were allowed to repeat the practice trial until the instructions were clear. The instructions and procedures were equal for the tasks with visually and phonologically similar items.

The experimental tasks were all serial order reconstruction tasks. First, a sequence of empty squares was presented. In the first square, a picture appeared for 2 s. and then disappeared. Then, the next picture appeared in the second square for 2 s. and disappeared. This procedure went on until the last picture had been presented. After the last picture had disappeared, all of the pictures reappeared on the bottom of the screen in a different position than the one they had been presented. The order in which the pictures reappeared was the same for the visually as for the phonologically similar tasks. For example, if the picture presented in the first position reappeared in the third position in the phonological similarity condition, the first picture of the visual similarity condition also reappeared in the third position. The sequence of squares remained empty on the screen and around the first square a bright yellow frame appeared, indicating that the child had to point out the picture that was presented in the first position. When the child pointed to a picture, the experimenter dragged that picture into the first square. This was done to keep the load due to motor (in)ability to a minimum and to make sure that the child reconstructed the sequences in the required temporal order. After the picture was dragged into the square, the experimenter immediately clicked on a button, making the yellow frame appear around the next square, indicating that the child had to point out the picture that was presented in the next position. This procedure was performed quickly.

When the experimenter clicked on the button, the previous picture disappeared from the square and reappeared on the bottom of the screen. This way we measured how well children could remember the whole serial order of the pictures, thus avoiding that the number of possible pictures to place in the square would decrease in the latter positions. If the pictures would remain in the squares, there would remain only one picture in the bottom of the screen to be placed in the last position and if the other pictures are placed correctly, the last picture would always be correct, even if the child did not actually know which picture belonged in the last position. If the participants did not know which picture belonged to a certain square, they had to guess. Although the children were not allowed to leave a square blank, they were always allowed to use a picture twice. In total, the administration of the tasks with practice and experimental trials took about 10–20 min per child.

### The dynamic latent state model

Our aims and hypotheses were addressed in a dynamic latent state model, or latent Markov model (Collins and Wugalter, 1992; Kaplan, 2008; Visser, 2011). The dependent variables (DV's) are the responses of the children on the six STM tasks, collected in a vector. These responses concern the reconstruction of the serial position of each item. The DV's are dichotomous, because each item was placed on either the correct (1) or incorrect (0) serial position within a task with a certain list length. These responses were predicted by a categorical latent variable. This latent variable consists of *discrete latent states*, which in our case were expected to represent the types of processes that drive the children's responses on the STM tasks. The latent states are assumed to differ in the way the responses are affected by two independent variables (and their interaction). These independent variables are *Similarity* (*S*), indicating whether the items of a task were phonologically similar or visually similar, and *List length* (*L*), indicating whether the task consisted of 3, 5, or 7 items to reconstruct the serial order.

Because the DV's are dichotomous, the regression model used has the form of a logit model for the probabilities *P*(*y* = 1|*x*, *S*, *L*). These are the probabilities of placing an item on the correct serial position (*y* = 1), conditional on a child being in latent state *x* and item coming from a task with Similarity *S* and List length *L*. The logit regression model has the following form:

$$\text{logit}P(y=1|x,S,L)=\beta_{0x}+\beta_{1x}S+\beta_{2x}L+\beta_{3x}S\times L^{1}.$$

The first parameter, β_0x_, is the intercept for latent state *x*. The second parameter, β_1x_, concerns the regression weights for latent state *x* for phonological and visual similarity. The third vector of parameters, **β**_2x_, concerns the regression weights for latent state *x* for list lengths 3, 5, and 7, and the fourth vector of parameters, **β**_3x_, concerns the regression weights for latent state *x* for the interactions between similarity and list length.

When we translate this latent variable model into the question whether one or more processes are involved in STM performance, we should explore the number of latent states that describes the variation in the data best. When only one latent state is sufficient to describe the variation in responses, this means that only one general process underlies STM performance in all the children. However, when multiple latent states are required to describe the variation in responses, the interpretation of the latent states depends on the effect of similarity in latent each state. A significant effect of Similarity (**β**_1x_, **β**_3x_) indicates that the correct scores on the phonologically similar task differ from the correct scores on the visually similar task. Specifically, because the phonologically similar tasks are the reference category (coded as 0), a positive effect indicates higher scores on the visually similar tasks than on the phonologically similar tasks (interpreted as the use of verbal processes) and a negative effect indicates lower scores on the visually similar tasks than on the phonologically similar tasks (interpreted as the use of visual processes). A non-significant effect of Similarity indicates that there is no difference in correct scores between the phonologically and visually similar tasks.

To illustrate the interpretation of the latent states using the effects of Similarity, suppose that two latent states are found. The first latent state is characterized by a significant positive similarity effect while the second is characterized by a significant negative similarity effect. In this case, the first latent state is interpreted as a subgroup of children using verbal STM processes, while the second is interpreted as a subgroup of children using visual STM processes. However, it is also possible that the two latent states show no significant similarity effect and only differ from each other in terms of overall performance. In that case, we can only conclude that none of the children show specific use of visual/verbal STM processes and that the children in one subgroup show higher scores on all the tasks than the children in the other subgroup.

**Figure 1:**
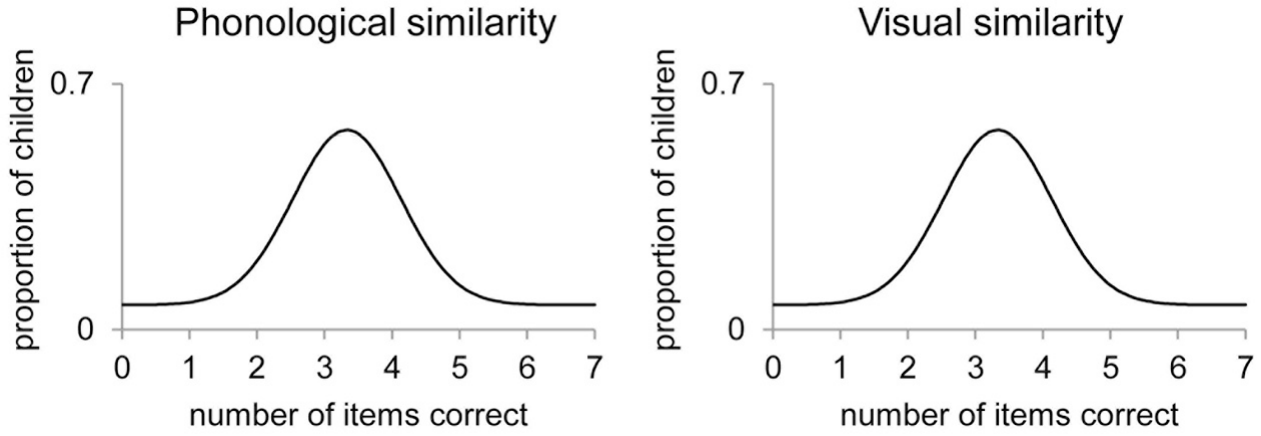
**Expected distribution of children′s responses when no specific verbal/visual STM processes are used**. Legend: The responses of all the children can be summarized by one distribution representing the memory process that is equally affected by phonological and visual similarity.

**Figure 2:**
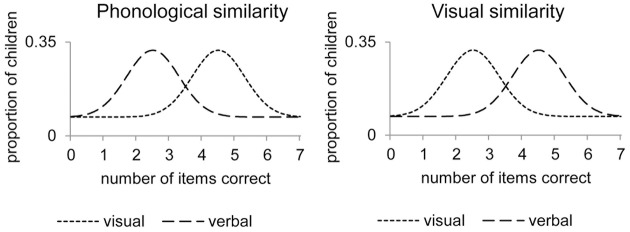
**Expected distribution of children′s responses when verbal (dashed) or visual (dotted) STM processes are used**. Legend: The responses of all the children are represented by a mixture of distributions reflecting the latent states. One distribution represents the responses of children using verbal STM processes (dashed lines). These children have lower means on the phonologically similar tasks than on the visually similar tasks. The other distribution represents the responses of children using visual STM processes (dotted lines), who have a lower number of items correct on the visually similar tasks than on the phonologically similar tasks.

Figure 1 illustrates the expected distribution of the responses of the children on a 7-items task in the case that no specific verbal/visual STM processes are found. The responses of all the children can be summarized by one distribution representing the memory process that is equally affected by phonological and visual similarity. Figure 2 illustrates the expected distributions of the responses of the children in the case that specific verbal/visual STM processes are found. The responses of all the children are represented by a mixture of distributions reflecting the latent states. One distribution represents the responses of children using verbal STM processes (dashed lines). These children have lower means on the phonologically similar tasks than on the visually similar tasks. The other distribution represents the responses of children using visual STM processes (dotted lines), who have a lower number of items correct on the visually similar tasks than on the phonologically similar tasks.

Furthermore, the effect of list length **β**_2x_ indicates the quantitative differences in performance when the children have to reconstruct the serial order of 3, 5, and 7 items. Increasing list length should lead to a relative decrease in performance. When list length increases, the distributions as illustrated in Figures 1, 2 are expected to shift to the left, showing lower means.

In the dynamic part of the model we investigated how children move from one latent state to another as list length increased from 3 to 5 items and from 5 to 7 items in order to address our question of how individual children change in the use of STM processes as the tasks get more difficult. The probability of belonging to a certain latent state *x* on a task with a particular list length is assumed to depend on the *initial state probabilities* and the *transition probabilities* (Vermunt et al., 2008). In our case, the initial state probabilities are represented as *P*(*x*3) and the transition probabilities as *P*(*x*5|*x*3) and *P*(*x*7|*x*5), where the subscripts refer to the list lengths of the tasks. Similar to the response probabilities, also these probabilities can be modeled with logit regression models; that is,

$$\begin{array}{l} \text{ logit}P(x3)=\gamma_{x3},\\ \text{logit}P(x5|x3)=\gamma_{x5x3},\\ \text{logit}P(x7|x5)=\gamma_{x7x5}. \end{array}$$

In these equations, the γ_*x*3_ parameters capture the overall probability of being in a certain initial state (on the tasks with 3 items). The γ_*x*5*x*3_ and γ_*x*7*x*5_ parameters capture the probability of moving to another state (switching) when list length increases from 3 to 5 and from 5 to 7 items, respectively, compared to the probability of staying in the same state (non-switching) when list length increases on the subsequent tasks.

More specifically, when the parameters of the transition probabilities are not significant (γ_*x*5*x*3_andγ_*x*7*x*5_ = 0) we can conclude that the probability of switching is equal to the probability of non-switching, that is, that the children can show any type of process on the subsequent tasks and therefore, that the type of process shown on one task does not change systematically across tasks with increasing list lengths. In contrast, if the type of process is stable across list lengths, the probability of switching should always be significantly smaller than the probability of non-switching (γ_*x*5*x*3_ and γ_*x*7*x*5<0_). To clarify even further how children move from one process to another, it is interesting to check whether the regression weights differ significantly across list lengths (γ_*x*5*x*3_ ≠ γ_*x*7*x*5_); that is, whether the transition probabilities change with increasing list length.

### Statistical analysis

We used the program Latent GOLD 4.5 (Vermunt and Magidson, 2005, 2008) to perform the analysis of our dynamic latent state model. First, we determined the number of latent states that represented the data best by fitting models with an increasing number of latent states and interpret the model that showed the best fit. The log likelihood of a model indicates the fit of the model to the data, with a lower value indicating a better fit (i.e., a smaller difference between the estimated model and the observed data). The number of parameters indicates the parsimony of the model. The balance between fit and parsimony of different models was estimated using the Bayesian Information Criterion (BIC, defined as −2 × log likelihood + number of parameters × ln(N)) and the Aikake Information Criterion 3 (AIC3, defined as −2 × log likelihood + 3 × number of parameters), which penalizes more for the number of parameters than the more commonly used AIC. The model with the lowest value on the information criteria indicates the best balance between fit and parsimony and is the one that should be interpreted. Next, the latent states of the model were interpreted by examining the effects of similarity and list length. Note that the interpretations and concurrent labels of the latent states were formulated by the authors based on the previously mentioned hypotheses.

The approach described until now actually corresponds to a latent class regression model. However, using a latent class regression model, we would be assuming that the latent states are stable, or static, across list lengths. Since we do not want to make this assumption, we used a dynamic latent state model, which links three latent class regression models (one for each task with a certain list length) to investigate the dynamics between the latent states across tasks.

To examine the moves from one process to another as list length increased, we examined the probabilities of belonging to a latent state on the 3-items tasks and the probabilities of moving to a latent state on the 5− and 7-items tasks, given the latent state on the previous task. The data structure and syntax for running the dynamic latent state model is included in Appendices B and C. Finally, we displayed the frequencies of subsequent latent states for the tasks with 3, 5, and 7 items in order to get an idea of the changes in the types of processes children use when the tasks get more difficult.

## Results

Table 1 shows the fit statistics of the dynamic latent state models that were fitted to our data. Increasing the number of latent states from one to two led to a substantive decrease in log likelihood, indicating that the model with two latent states showed a better fit to the data. The decrease in the BIC and AIC3 values confirmed that the model with two latent states showed a better balance between fit and parsimony. This finding indicates that the model with one latent state, assuming that all the responses are represented by one process, can be rejected. In order to find the number of latent states that represent the data best, more models with an increasing number of latent states were fitted until the lowest values of the BIC and the AIC3 were reached. Increasing the number of latent states up until four led to a decrease in the BIC and AIC3 values. However, increasing the number of latent states to five led to an increase of the BIC and AIC3 values. Therefore, the effects of similarity and list length were interpreted for four latent states.

**Table 1:** **Fit statistics of the five fitted models**.

**Model**	**Log likelihood**	**Number of parameters**	**BIC**	**AIC3**
One latent state	−3558.57	6	7149.22	7135.14
Two latent states	−3197.00	17	6484.91	6445.01
Three latent states	−3056.87	32	6284.85	6209.74
Four latent states	−2979.67	51	6232.04	6112.34
Five latent states	−2959.59	74	6314.87	6141.18

The overall effect of Similarity was significant, Wald's χ^2^(4) = 264.25, *p* < 0.001, and it differed over the latent states, Wald's χ^2^(3) = 260.85, *p* < 0.001. There was also a significant effect of List length, Wald's χ^2^(8) = 189.23, *p* < 0.001, which differed over the latent states, Wald's χ^2^(6) = 169.78, *p* < 0.001. The overall interaction between Similarity and List length was also significant, Wald's χ^2^(8) = 38.08, *p* < 0.001, and differed over the latent states, Wald's χ^2^(6) = 33.74, *p* < 0.001, indicating that the effect of similarity differed across the tasks with increasing list length. The parameter estimates and standard errors of the effects on the probability of a correct score in each latent state are given in Appendix D.

In order to interpret each latent state we used the estimated parameters to calculate the probabilities of a correct response on the phonologically and visually similar tasks with each of the list lengths (see Table 2). The latent states were labeled “verbal” or “visual” only if the effect of similarity was significant for that particular list length. If the effect of similarity was not significant, the labels referred merely to the overall probability of a correct response (“high,” “low,” and “lower”), meaning that the probability of a correct score was equal for the visually and the phonologically similar tasks. The parameter estimates and standard errors for the effect of visual similarity compared to phonological similarity per list length for each latent state are given in Appendix E.

**Table 2:** **Probabilities of a correct score, interpretations, and characteristics of each latent state**.

	**Overall**	**Phonological similarity**	**Visual similarity**	**Inter-pretation**	***N***	**Age range**
**3 ITEMS TASK**						
State 1	0.67	0.36	0.97	Verbal	23	5;2–12;7
State 2	0.65	0.99	0.30	Visual	18	4;9–12;4
State 3	0.99	0.99	0.99	High	159	5;0–12;8
State 4	0.30	0.37	0.23	Low	10	6;4–12;3
**5 ITEMS TASK**						
State 1	0.76	0.53	0.99	Verbal	46	5;1–12;7
State 2	0.79	0.99	0.58	Visual	43	5;3–12;4
State 3	0.99	0.99	0.99	High	74	5;0–12;8
State 4	0.45	0.42	0.47	Low	47	4;9–12;3
**7 ITEMS TASK**						
State 1	0.82	0.64	0.99	Verbal	22	5;2–12;4
State 2	0.77	0.99	0.54	Visual	32	5;7–12;4
State 3	0.46	0.48	0.43	Low	111	5;0–12;8
State 4	0.18	0.17	0.19	Lower	45	4;9–12;3

Table 2 shows that in the 7-items tasks, for instance, children in state 1 had a probability of obtaining a correct score of 0.64 on the phonologically similar task and a probability of 0.99 on the visually similar task. The probability of a correct response was significantly higher for the visually similar than for the phonologically similar task (see Appendix E, difference parameter = 8.41, *SE* = 0.59, *p* < 0.001). This pattern was also found in states 1 on the tasks with 5 and with 3 items. These differences can be interpreted as verbal processing being involved in the responses of children in states 1 on all the tasks. These states were therefore labeled “verbal.” Note, however, that these three verbal states do not necessarily contain the same children. In states 2, the effect of similarity was also significant on all the tasks, but negative, indicating lower scores on the visually similar tasks than on the phonologically similar tasks. States 2 were therefore labeled “visual.” The effects of similarity were not significantly different in states 3 and 4 on all the tasks. Therefore, we labeled these states according to their overall performance, which depended on the list lengths of the tasks. For instance, states 3 on the 3- and 5-items tasks showed high overall performance (probability correct of 0.99), but on the 7-items tasks state 3 showed low overall performance (probability correct of 0.46).

Furthermore, the age ranges in Table 2 show that the differences between the latent states were not related to age differences. The children in the latent states found with the 3-items tasks showed mean ages between 8;5 and 9;0 years, which did not differ from each other, *F*_(3, 206)_ = 0.272, *p* = 0.85. The children in the latent states found with the 5-items tasks showed mean ages between 8;5 and 9;1 years, which did not differ from each other either, *F*_(3, 206)_ = 0.684, *p* = 0.56. Also, the mean ages of the children in the latent states found with the 7-items tasks, ranging between 8;0 and 9;0 years, did not differ from each other, *F*_(3, 206)_ = 1.715, *p* = 0.17.

Further inspection of Table 2 shows that overall performance did not decrease with increasing list lengths in the “verbal” and “visual” states. The probability of a correct score sometimes even increases with increasing list length, which may seem strange, but actually is a consequence of the relative nature of probabilities. It makes more sense in this case to express the scores in absolute correct scores. On the 3-items tasks, the children in the “verbal” state scored on average 2 items correct and the children in the “visual” state also scored on average 2 items correct (overall probabilities of 0.67 and 0.65). On the 5-items tasks, the children in the “verbal” state scored on average 4 items correct and the children in the “visual” state also scored on average 4 items correct (overall probabilities of 0.76 and 0.79). On the 7-items tasks, the children in the “verbal” state scored on average 6 items correct and the children in the “visual” state scored on average 5 items correct (overall probabilities of 0.82 and 0.77). This confirms that the differences in responses between these states are not quantitative differences, because the children in both states commit only 1 or 2 errors on each list length. Rather, the differences are influenced by the type of similarity, indicating that these children show qualitative differences in responses.

The opposite was found for the children in the remaining states showing quantitative, but not qualitative differences. On the 3-items tasks the children from the “high” state scored on average 3 item correct, whereas children from the “low” states scored on average 1 item correct (overall probabilities of 0.99 and 0.30). On the 5-items tasks, the children from the “high” state scored on average 5 items correct, whereas children from the “low” state scored on average 2 items correct (overall probabilities of 0.99 and 0.45). Finally, on the 7-items tasks the children from the “low” state scored on average 3 items correct, whereas children from the “lower” state scored on average 1 item correct (overall probabilities of 0.46 and 0.18).

With respect to the dynamic part of the model, the initial state probabilities were significant, Wald's χ^2^(3) = 154.30, *p* < 0.001. The transition probabilities were also significant, Wald's χ^2^(24) = 688.40, *p* < 0.001, and differed across tasks with specific list lengths, Wald's χ^2^(12) = 74.42, *p* < 0.001. These latter findings indicate that children do not consistently move to another state or stay in the same state on subsequent tasks with increasing list lengths. Moreover, the transition probabilities differed not only across list lengths, but also across origin states, Wald's χ^2^(9) = 54.03, *p* < 0.001.This means that the specific switches from one latent state to another change when the list lengths increase from 3 to 5 items compared to when the list lengths increase from 5 to 7 items. The estimated parameters and standard errors for the effects on the transition probabilities (the probabilities of moving to each latent state from each previous latent state) are given in Appendix F.

Finally, for the latent states to which each child was assigned the frequencies of subsequent latent states on all the STM tasks were analyzed. Table 3 shows the sequences of latent states on the tasks with 3, 5, and 7 items with frequencies larger than 4 and the age ranges of the children showing each sequence of latent states (the remaining sequences of latent states with frequencies smaller than 4 were made by 44 children in total, not shown in Table 3). As can be seen, children did not consistently belong to the “verbal” or “visual” states across list lengths, but most of them did show the use of specific verbal/visual STM processes in at least one of the list lengths. Only seven children changed from verbal to visual STM processes when the tasks increased from list lengths of 5–7 items. None of the changes in STM processes across tasks were related to age, *F*_(13, 196)_ = 0.732, *p* = 0.73.

**Table 3:** **Frequencies of latent states sequences on the STM tasks and age ranges**.

**3 items**	**5 items**	**7 items**	**Frequency**	**Age range**
High	High	Low	35	5;0–12;8
High	Visual	Low	21	5;3–12;4
High	Verbal	Low	17	5;1–11;8
High	High	Visual	17	5;7–11;9
High	Low	Low	14	7;4–11;8
High	Low	Lower	12	5;5–12;1
High	High	Verbal	11	6;2–11;0
Verbal	Low	Lower	8	6;7–12;1
High	Verbal	Visual	7	7;3–11;3
High	Verbal	Verbal	6	5;2–10;2
High	Visual	Visual	6	6;3–11;7
High	Verbal	Lower	6	5;9–11;6
High	Visual	Lower	6	6;9–11;8

## Discussion

This study aimed at unraveling verbal and visual STM processes and examining the changes children show in the use of STM processes across tasks with increasing difficulty. We addressed our aim by designing our experiments in a way that allowed us to detect the types of processes involved in STM tasks. By defining a dynamic latent variable model investigating different latent states and at the same time allowing the latent states to differ across list lengths, we could investigate the number of STM processes involved in children's responses as well as how individual children move from one type of process to another as list length increases.

Our results showed that different processes could be distinguished in the responses of children on STM tasks with increasing list lengths. The differences in the responses of two small states were qualitative differences indicating verbal and visual processes and the differences in two larger states were quantitative differences in overall performance. The quantitative differences in overall performance were strongly related to list length. When children had to reconstruct the serial order of tasks with 3 and 5 items, there was a considerably large group that performed nearly perfect (“high” states). However, when they had to reconstruct the serial order of tasks with 7 items, the overall performance of the children dropped, resulting in the “low” and “lower” states. In contrast, the qualitative differences between children in verbal and visual processes were consistently present on all the tasks. In other words, on every list length there was a group of children who performed worse when the pictures were visually similar but not when the pictures were phonologically similar (indicating the use of visual STM), as well as a group of children who performed worse when the pictures were phonologically similar but not when they were visually similar (indicating the use of verbal STM). The finding that our data was represented best by these multiple states showing quantitative as well as qualitative differences in performance supports the modality-specific view of STM.

Whether or not different processes were involved in the responses on the STM tasks could be revealed using an analysis of (dynamic) latent states. In comparison, if we had used the more common analysis of variance (ANOVA), we would be assuming that the responses of all the children are appropriately summarized by one distribution. As such, the effects of similarity would not have become apparent in our results, because the variation in responses due to similarity is systematic only in a small group of children (belonging to the “verbal” and “visual” states of the current analysis). The systematic variation in the responses of this small group of children would have been considered as measurement error when analyzing the sample as a whole. Therefore, we would only have found differences in overall performance, which would have seemed to support the functionally equivalent view of STM. However, this approach becomes problematic when the variation in the responses of a sample is not appropriately summarized by one distribution, as was the case in our study and as will probably often be the case, especially when a sample consists of children of different ages. Instead, considering the possibility that the children's responses are driven by multiple processes, or latent states, provided us with valuable information that would otherwise have been lost.

When we shift our focus to the individual children, our results showed that children move from one type of processing to another as list length increases. Although the most frequent move was from higher to lower overall performance, many of the remaining moves contained verbal or visual processes in at least one task with a certain list length. However, in order to obtain a complete view of how the children switched between processes, we should interpret the processes as a sequence across all list lengths, as shown in Table 3. For instance, there was a group of children who moved from high performance on the 3-items tasks to visual performance on the 5-items tasks, to low performance on the 7-items tasks (“high-visual-low”), and a group of children showing the sequence “high-verbal-low.” These children may have used visual and verbal processing, respectively, on the 3-items and the 7-items tasks as well, but the interference of similarity did not become apparent in their responses because reconstructing the serial order of 3 items might have been be too easy and reconstructing the serial order of 7 items too difficult. In such cases, the difficulty related to list length might have masked the interference of similarity. This could also be the case for the group of children showing the sequence “high-visual-lower” and “high-verbal-lower.” In contrast, for the groups showing the sequence “high-high-visual” and “high-high-verbal,” reconstructing the serial order of 7 items might not have been too difficult, but challenging enough for the interference of the similarity to become apparent in their responses. The same may apply for the groups showing the switches “high-verbal-verbal” and “high-visual-visual” (see Table 3). Based on the moves we found, we argue that even though STM processes can be considered to be modality-specific, that there are few children who will consistently show either verbal or visual processing, because task conditions such as difficulty also play an important role in performance.

Another possible explanation why children do not consistently show either verbal or visual processing, is that verbal and visual processing are combined within a single task, as previous studies with adults have shown (Logie et al., 2000; Saito et al., 2008). To unravel such a combination of STM processes it would be useful to examine the types of errors made on each serial position. When children apply verbal processing and use additional visual processing when the task gets more difficult, this should be reflected by higher scores on visually similar items than phonologically similar items in the first positions of the sequence and the reversed effect in the last positions of the sequence. When children apply visual processing and use additional verbal processing, this should be reflected by higher scores on phonologically similar items than visually similar items in the first positions of the sequence and the reversed effect in the last positions of the sequence. Hypotheses like these would be very useful to gain more insight in the combination of verbal and visual processing and could also be tested with latent class models.

To summarize, our results show a distinction in verbal and visual process states as well as high and low performance states. Moreover, the verbal and visual process states were consistently present in STM tasks with increasing list lengths. The differences within children in terms of switching indicate that most children show verbal or visual processing in at least one list length, but they do not consistently show either verbal or visual processing across all list lengths. In conclusion, we claim that STM processes can only be investigated well by taking the influence of task conditions into consideration. Moreover, this study shows that these tasks conditions are also likely to interact with individual differences in responses on memory tasks driven by specific processes. In order to catch these complex systems of intertwining players, dynamic latent variable models in combination with a thoughtfully set up design are of great value.

## Author contributions

Conception and design of the work; Gabriela V. Koppenol-Gonzalez, Samantha Bouwmeester. Acquisition of the data; Gabriela V. Koppenol-Gonzalez. Analysis; Gabriela V. Koppenol-Gonzalez, Samantha Bouwmeester, Jeroen K. Vermunt. Interpretation of the data; Gabriela V. Koppenol-Gonzalez, Samantha Bouwmeester, Jeroen K. Vermunt. Drafting the article; Gabriela V. Koppenol-Gonzalez. Revising the article critically for important intellectual content; Samantha Bouwmeester, Jeroen K. Vermunt. Final approval of the version to be published. Gabriela V. Koppenol-Gonzalez, Samantha Bouwmeester, Jeroen K. Vermunt.

### Conflict of interest statement

The authors declare that the research was conducted in the absence of any commercial or financial relationships that could be construed as a potential conflict of interest.

## Appendix B

### Data structure for running the dynamic latent state model in latent gold 4.5

**Table:**

**ID**	**Timeid**	**Listlength57**	**Listlength357**	**Similarity**	**Score**
1	1	.	1	Phonological	1
1	1	.	1	Phonological	1
1	1	.	1	Phonological	1
1	1	.	1	Visual	1
1	1	.	1	Visual	1
1	1	.	1	Visual	1
1	2	1	2	Phonological	1
1	2	1	2	Phonological	1
1	2	1	2	Phonological	1
1	2	1	2	Phonological	1
1	2	1	2	Phonological	1
1	2	1	2	Visual	1
1	2	1	2	Visual	1
1	2	1	2	Visual	1
1	2	1	2	Visual	1
1	2	1	2	Visual	1
1	3	2	3	Phonological	1
1	3	2	3	Phonological	0
1	3	2	3	Phonological	0
1	3	2	3	Phonological	1
1	3	2	3	Phonological	1
1	3	2	3	Phonological	1
1	3	2	3	Phonological	1
1	3	2	3	Visual	1
1	3	2	3	Visual	0
1	3	2	3	Visual	0
1	3	2	3	Visual	0
1	3	2	3	Visual	0
1	3	2	3	Visual	0
1	3	2	3	Visual	0
2	1	.	1	Phonological	1
2	1	.	1	Phonological	0
…	…	…	…	…	…

*., missing; …, etc*.

## Appendix C

### Syntax for running the dynamic latent state model in latent gold 4.5

variables

     caseid ID;

     timeid timeid;

     dependent Score;

     independent Similarity nominal, Listlength57 nominal,

Listlength357 nominal;

     latent

        State nominal dynamic 4;

   equations

     State[=0] <− 1;

     State <− (~tra) 1 |Listlength57 State[−1];

       Score <−1 |State + Similarity |State + Listlength357

|State + Similarity Listlength357|State;

## Appendix D

### Parameter estimates and standard errors for the effects on the probability of a correct score for each latent state

**Table:**

	β**_0_*X***	β**_1*X*_*S***	β**_2*X*_*L***	β**_3*X*_*L*** × ***S***
**PARAMETERS 3 ITEMS**				
State 1	1.39 (0.10)^***^	2.75 (0.23)^***^	−1.36 (0.37)^***^	−0.75 (0.40)
State 2	1.85 (0.23)^***^	−3.81 (0.47)^***^	−0.39 (0.52)	−0.36 (0.56)
State 3	2.63 (0.28)^***^	0.30 (0.53)	1.81 (0.96)	1.37 (0.90)
State 4	−0.43 (0.08)^***^	−0.05 (0.12)	−0.01 (0.22)	−0.29 (0.22)
**PARAMETERS 5 ITEMS**				
State 1	1.39 (0.10)^***^	2.75 (0.23)^***^	−0.62 (0.32)	−0.71 (0.32)^*^
State 2	1.85 (0.23)^***^	−3.81 (0.47)^***^	1.30 (0.46)^**^	−0.85 (0.48)
State 3	2.63 (0.28)^***^	0.30 (0.53)	3.63 (0.72)^***^	−0.96 (0.72)
State 4	−0.43 (0.08)^***^	−0.05 (0.12)	0.66 (0.17)^***^	0.15 (0.12)
**PARAMETERS 7 ITEMS**				
State 1	1.39 (0.10)^***^	2.75 (0.23)^***^	1.98 (0.26)^***^	1.46 (0.29)^***^
State 2	1.85 (0.23)^***^	−3.81 (0.47)^***^	−0.92 (0.86)	1.21 (0.88)
State 3	2.63 (0.28)^***^	0.30 (0.53)	−5.45 (0.61)^***^	−0.41 (0.53)
State 4	−0.43 (0.08)^***^	−0.05 (0.12)	−0.65 (0.24)^**^	0.14 (0.17)

*The effects of similarity are given for visual similarity, for phonological similarity the effects have the same value with a reversed sign*.

*** p < 0.001,

** p < 0.01, and

* *p < 0.05*.

## Appendix E

### Parameter estimates and standard errors for the effects of visual similarity compared to phonological similarity per list length for each latent state

**Table:**

	**Visual similarity**	**Interpretation**
**PARAMETERS 3 ITEMS**		
State 1	3.99 (1.07)^***^	Verbal
State 2	−8.34 (1.10)^***^	Visual
State 3	3.33 (2.59)	High
State 4	−0.67 (0.61)	Low
**PARAMETERS 5 ITEMS**		
State 1	4.08 (0.72)^***^	Verbal
State 2	−9.32 (0.24)^***^	Visual
State 3	−1.34 (1.78)	High
State 4	0.21 (0.21)	Low
**PARAMETERS 7 ITEMS**		
State 1	8.41 (0.59)^***^	Verbal
State 2	−5.21 (2.59)^*^	Visual
State 3	−0.22 (0.15)	Low
State 4	0.18 (0.33)	Lower

*** p < 0.001, and

* *p < 0.05*.

## Appendix F

### Parameter estimates and standard errors for the effects on the initial state probabilities and transition probabilities

**Table:**

	**Current state 1**	**Current state 2**	**Current state 3**	**Current state 4**
**INITIAL STATE**				
γ_*x*3_	−0.21 (0.24)	−0.45 (0.21)^*^	1.73 (0.14)^***^	−1.07 (0.29)^***^
**PREVIOUS STATE 1**				
γ_*x*5*x*3_	0	0.37 (0.94)	0.20 (1.13)	1.24 (0.83)
γ_*x*7*x*5_	0	−0.05 (0.55)	1.10 (0.44)	0.01 (0.61)
**PREVIOUS STATE 2**				
γ_*x*5*x*3_	−0.35 (0.74)	0	−0.43 (0.78)	−0.03 (0.67)
γ_*x*7*x*5_	−1.83 (1.12)	0	1.42 (0.56)^*^	0.39 (0.92)
**PREVIOUS STATE 3**				
γ_*x*5*x*3_	−0.47 (0.22)^*^	−0.55 (0.23)^*^	0	−0.86 (0.26)^***^
γ_*x*7*x*5_	−1.25 (0.38)^**^	−0.66 (0.31)^*^	0	−5.96 (1.25)^***^
**PREVIOUS STATE 4**				
γ_*x*5*x*3_	−0.64 (0.92)	−4.71 (1.11)^***^	−1.73 (1.16)	0
γ_*x*7*x*5_	−6.65 (0.49)^***^	−6.57 (0.52)^***^	−0.39 (0.77)	0

*** p < 0.001,

** p < 0.01, and

* *p < 0.05*.
